# Atomization of Cocoa Honey Using Whey Protein Isolate to Produce a Dry Formulation with Improved Shelf Life for Industrial Application

**DOI:** 10.3390/foods12234269

**Published:** 2023-11-26

**Authors:** Christiano Pedro Guirlanda, Izabela Dutra Alvim, Jacqueline Aparecida Takahashi

**Affiliations:** 1 Food Science Graduate Program, Department of Food Science, College of Pharmacy, Universidade Federal de Minas Gerais, Av. Antônio Carlos, 6627, Belo Horizonte 31270-901, MG, Brazil; cpguirlanda@gmail.com; 2 Institute of Food Technology, Cereal and Chocolate Technology Center, Av. Brasil 2880, Campinas 13070-178, SP, Brazil; izabela@ital.sp.gov.br; 3 Department of Chemistry, Exact Sciences Institute, Universidade Federal de Minas Gerais, Av. Antônio Carlos, 6627, Belo Horizonte 31270-901, MG, Brazil

**Keywords:** cocoa honey, residues, atomization, self-life increasing, circular economy

## Abstract

Cocoa honey, a by-product obtained during the processing of cocoa, is a juice rich in pectin, organic acids, minerals and phenolic compounds with antioxidant properties. Fresh cocoa honey is quickly fermented due to its high content of reducing sugars, such as fructose and glucose, which limits its shelf life. Currently, cocoa honey is only commercialized in frozen form, as logistical challenges prevent the wide distribution or export of this by-product for applications in the market of sweets, jellies, beverages, confectionery, and nutraceutical foods among others. Spray-drying technology is a viable prospect for the large-scale stabilization of products such as cocoa honey, with less heat exposure compared to other conventional drying methods. This work aimed to evaluate the efficacy of drying adjuvants for a rapid removal of the water present in cocoa honey via atomization, since this process minimizes the effects of glass transition temperature (Tg) related to materials with high sugar contents. Physical parameters such as the moisture content, hygroscopicity, particle size, and yield of the products obtained were determined. Cocoa honey presented 85.3 ± 0.20 g/100 g of moisture. The formulations successfully decreased moisture content, which was lower than 11.72 ± 0.08 g/100 g in the formulations. Water activity ranged between 0.1464 ± 0.0043 and 0.1562 ± 0.029, with no significant difference between the formulations. The hygroscopicity of cocoa honey powders ranged from 29.29 to 29.87 g of water/100 g of cocoa honey. The combination of 20% maltodextrin and 1% whey protein isolate (WPI) led to the best yield, resulting in a free-flowing powder as the final product. On the other hand, the formulation composed of maltodextrin and whey protein isolate in the ratio of 29:1, respectively, led to the most stable product, with less loss of phenolic compounds during the drying process (6.04%). Regarding particle diameter, 90% of the accumulated distribution did not exceed 57 μm. The greatest dispersion of particles occurs in the Ma20W10 formulation with a span of 2.72, inferring greater variation in size between small (7.01 ± 0.06 μm), medium (18.25 ± 0.37 μm), and large (56.65 ± 1.17 μm) particles. The use of whey protein isolate as an adjuvant proved to be an efficient drying process in the production of cocoa honey powder, and was also advantageous for enriching the nutritional content of the final product due to its protein origin. Furthermore, the combination of spray-drying technology and the use of whey protein isolate as adjuvant led to a free-flowing cocoa honey powder with an adequate particle size and benefits in terms of shelf-life extension, providing new opportunities for the commercialization of cocoa honey as an ingredient for the food industry, with benefits for the circular economy.

## 1. Introduction

Cocoa cultivation occupies a prominent place in the world economy because this fruit generates important products for various segments such as confectionery, dairy, and alcoholic beverages, as well as the pharmaceutical, cosmetic, and perfumery sectors [1,2]. The emergence of new demands, such as fine chocolates, as well as the description of various functional effects of biomolecules present in cocoa, should drive the continued growth of cocoa production in the coming years [3]. However, this growth requires urgent improvements in the structure of the production chain, which generates various types of waste such as fruit peels, pulp, and residues from the roasting and grinding steps. The sustainable expansion of cocoa production requires the development of processes that use by-products and waste generated in the production chain [4,5].

Cocoa pulp is rich in important nutrients, and is tasty and recognized as a healthy and functional food with antioxidant benefits [6]. Cocoa pulp can be pressed to provide a by-product known as cocoa honey, a natural juice important for the circular economy due to its enormous industrial commercial potential [7,8]. Cocoa honey has been sold in the form of frozen juice, mainly in Central and South America, where it is also used to produce alcoholic beverages, honeydews, vinegar, and jellies.

However, only a small part of this juice is consumed, as the shelf life of fresh cocoa honey is very short. Most of the cocoa honey is lost during cocoa processing, as the high sugar content makes this byproduct very susceptible to fermentation. Therefore, the development of processes to stabilize this juice is essential to increase its shelf life before its commercialization [7]. The use of heating stabilization techniques, such as pasteurization, are widely used in food industry, but, in some situations, it can generate losses in nutritional quality, a reduction in bioactive compounds, significant organoleptic alterations, and browning due to the Maillard reaction. These problems limit the application of certain processes to enable the commercialization of fresh cocoa honey. Therefore, an alternative would be the production of a powder from cocoa honey, which could be recomposed for the production of juices or used as an ingredient with multiple applications in the food industry. This type of processing requires the use of adjuvants to avoid the formation of a highly hygroscopic and sticky product, a common problem in the preparation of powdered fruit juices [9]. In this context, the objective of this work was to dry cocoa honey via atomization, using adjuvants, obtain a free-flowing powder product, and characterize the stability of this product.

## 2. Materials and Methods

### 2.1. Materials

Cocoa honey (*n* = 3) was kindly provided by a producer (Ilhéus, Bahia, Brazil). The adjuvants maltodextrin (Mor-Rex 1910, dextrinization grade 10, Ingredion Mogi Guaçu, São Paulo, Brazil), methocel (E19, Hydroxypropyl methyl cellulose, Food Grade, Dow, Santo Amaro, São Paulo, Brazil), and whey protein isolate (WPI, Fonterra, Taranaki, New Zealand) were purchased commercially. The reagents used were of analytical grade. All experiments were carried out in triplicate.

### 2.2. Preparation of Formulations Using Maltodextrin, Methocel, and WPI as Excipients

To obtain the formulations (Ma30, Ma15Me15, Ma29W1, Ma20W10, and Ma15Me10W5), the powdered excipients (maltodextrin, methocel, and WPI) were weighed and added to the fresh cocoa honey following the proportions presented in Table 1. The percentages of excipients were calculated considering the percentage of solid materials present in cocoa honey, and were chosen to reach a maximum of 30% dm in the formulation. This value is commonly respected in preparations using fruit juices to avoid negative impact of the wall material on the physicochemical characteristics and acceptability of the product [10].

To obtain the powder formulations, the mixtures containing cocoa honey and the carriers were dehydrated in a spray dryer (B290, Buchi, Flawil, Switzerland) configured with a double fluid atomizer nozzle with a 0.7 mm hole. The spray pressure was maintained at 0.75 bar and the airflow at 600 L/min [11]. The inlet temperature of the dryer was 180 °C and the output was maintained at 85 ± 3 °C by controlling the feed flow. The percentage yields of the processing were determined by the mass of the sample collected in the collector and in the cyclone of the equipment at the end of each process, in relation to the total solids present in the initial formulation. For each formulation, three processes were performed, and the resulting powder preparations were packed in plastic packaging, covered with a lid, and stored in a desiccator (25 ± 3 °C) until the characterization stage.

#### Storage of Formulations

Half of the preparation’s content was individually transferred to plastic vials, sealed, and stored under refrigeration at 4 °C during 6 months to evaluate the effect of storage on the stability of the formulations. Cocoa honey powder preparations were characterized regarding moisture content, particle size, water activity, hygroscopicity, morphology, and total phenolic compound content immediately after being prepared and again after 6 months of storage under refrigeration.

### 2.3. Moisture Determination

The moisture of the cocoa honey, carriers, and formulations was determined through the gravimetric method according to AOAC methodology [11]. A total of 1 g of the dried formulations was weighed in triplicate and deposited in a previously weighed aluminum capsule. Drying was performed in an oven at 105 °C overnight until constant weight and the calculation of each determination was made according to the Equation (1).

Equation (1) Determination of moisture content

(1) $$Moisture\;(\%)=\left(\frac{M1-M2}{M2}\right)\times 100$$

where *M*1 = mass of total sample and *M*2 = mass of dry sample.

The average diameter and particle size distribution of the samples were determined through laser diffraction in a LA-950 V2 diffractometer (Horiba, Irvine, CA, USA), using absolute ethanol as a dispersion medium. The powder formulations were mixed with absolute ethanol and sonicated for 15 s to ensure the dispersion of the particles. The preparations were added to the diffractometer module until the concentration reached transmittance levels suitable for reading [12]. The preparations were read 6 times.

### 2.4. Water Activity Determination

The water activity (aW) of the samples was determined in triplicate at 25.0 ± 0.5 °C, using a hygrometer Aqualab model 4TEV (Decagon Devides Inc., Pullman, EUA) [12]. Water activity ranged from 0 (no water available) to 1 (100% water available).

### 2.5. Hygroscopicity Determination

The hygroscopicity of the formulations was determined according the methodology described by Cai and Corke [13] with modifications. A total of 1 g of each sample was weighed in triplicate and stored at 25 °C in the presence of saturated solution of NaCl 75.3% RH (relative humidity). After 7 days, weight gain due to moisture adsorption was recorded and expressed in the form of “g of adsorbed moisture/100 g of sample”.

### 2.6. Study of the Morphological Characteristics of the Formulation

The morphological aspect of the powdered formulations was determined using optical light microscopy model BX41 (Olympus, Tokyo, Japan). A digital camera (Q-Color3, Olympus, Japan) was adapted to the microscope for image capture [12]. A small amount of each powdered sample was placed on a microscope blade, and a drop of mineral oil was added to disperse the material. Then, the blade was covered with a slide and the material was observed on the microscope.

### 2.7. Total Phenolic Compounds Determination

Total phenolic compounds were determined through colorimetric method based on the reduction in phosphomolybdic and phosphotungstic acid in alkaline solution using Folin–Ciocalteu reagent (Sigma-Aldrich, São Paulo, Brazil). to produce a blue-colored compound. Color intensity is proportional to the number of phenol groups present in the molecules. A total of 2 mg of the material was mixed with 1 mL of distilled water, 0.5 mL of the Folin–Ciocalteu reagent, and 2 mL of 20% sodium bicarbonate. The mixture was vigorously stirred and incubated for 25 min at 40 °C. Total phenolic compounds were determined using a UV-vis spectrophotometer, and the absorbance was measured at 765 nm. The results were expressed in milligrams of gallic acid equivalent per gram of dry powder (mg GAE/g) [14].

### 2.8. Statistical Analysis

The experiments were carried out in triplicate, and the results of the analyses were treated statistically using analysis of variance (ANOVA), followed by comparison of means using the Tukey’s *t* test using the Minitab Statistical Software 20 version with a 95% confidence level. The results were expressed as mean ± standard deviation.

## 3. Results and Discussion

### 3.1. Effect of the Carriers’ Adjuvants Maltodextrin and Methocel in Cocoa Honey Atomization

Cocoa honey has, in its composition, low-temperature-glass-transition sugars such as glucose (Tg 31 °C), sucrose (Tg 62 °C), and fructose (Tg 5 °C), components that contribute to the high viscosity of cocoa honey. Consequently, it is hard to transform this viscous juice into powder, as the thermal plasticization of sugars with a low glass transition temperature makes the drying process difficult. This effect occurs when the temperature during processing rises by 10 to 20 °C above the glass transition temperature of the product. Low Tg values of fructose, as in cocoa honey, are influenced by the plasticizing capacity of water. The variation is in accordance to the concentration of water present in the solution; the greater the amount of water, the lower the Tg [15,16,17]. To enable the atomization of cocoa honey, the carrier adjuvants maltodextrin, methocel, and WPI were used as adjuvants in the process, in different proportions (Table 1). These carriers modify the characteristics of the material, forming an outer layer that alters the surface stickiness of the particles due to the transformation of the vitreous state, improving the performance of the drying process and, consequently, increasing the yield of the product. After drying, the powders were recovered from the equipment chamber via mechanical scraping, packed in dry bottles, weighed, and stored in a desiccator.

**Figure 1:**
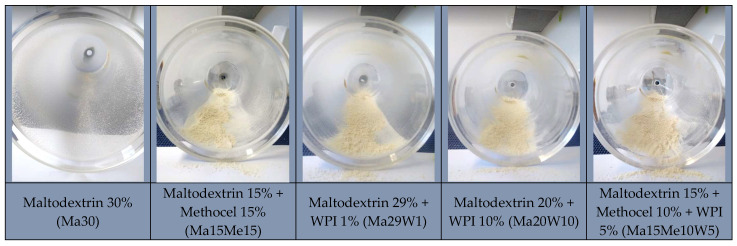
Aspect of the formulations containing cocoa honey and excipients maltodextrin, methocel, and WPI, in different combinations, recovered in the drying chamber of the spray dryer.

The adjuvants and their concentrations in the formulations were chosen in accordance with the literature, taking into consideration several parameters such as cost, interaction with the starting material, safety, solubility, effect and, most importantly, absence of flavor. The maximum concentration was established in 30%, taking into consideration the final cost of the product as an important factor for further product development. The first adjuvant tested was maltodextrin, a substance widely used in the food industry as carrier due to its high solubility in water, low viscosity, and mild flavor. Furthermore, it is colorless in solution, offers protection against oxidation when used as a wall material, and has a low cost [10,18]. However, the Ma30 formulation, prepared with 30% maltodextrin, was retained in the chamber wall even after scraping, and the yield of the atomization was practically zero. This result indicates that this carrier was not efficient in reducing the viscosity of cocoa honey in order to avoid deposition on the chamber wall. The appearance of the atomization products of cocoa honey processed with different excipients, recovered in the drying chamber, is shown in Figure 1.

The concentration of carriers used in spray drying should not influence the physicochemical properties of the dried material [19,20]. It was found that a high concentration of maltodextrin increases the viscosity of the formulation, which ends up reducing the final powder yield and increasing the particle size [21]. The adhesion of the product to the internal wall of the dryer and agglomeration of particles, although common in spray drying, reduces the process yield and generates a powder of low commercial quality [22]. To avoid increasing the amount of maltodextrin in the product, a formulation containing 15% maltodextrin and 15% methocel (Ma15Me15) was prepared, as the latter has been successfully employed to improve the physicochemical properties and increase yield of the product in combination with maltodextrin [23]. In fact, the presence of methocel made it possible to recover the Ma15Me15 formulation in powder form (30.25% yield), demonstrating that the combination of the carriers maltodextrin and methocel generates a free-flowing product. Product yield was defined as the ratio between the mass of the dry product collected after drying and the weight of the total solids of the formulation.

### 3.2. Effect of Whey Protein Isolate on Cocoa Honey Atomization

To improve the fluidity of the formulation, the effect of whey protein isolate (WPI) on the drying of cocoa honey was also studied. WPI itself is a wall material that has an excellent film-forming capacity, functional properties, and high solubility, and has been reported to be a useful carrier when mixed with maltodextrin [24,25]. The combination of these carriers reduces inter-surface interaction while furnishing good surface coating properties and increased glass transition temperature. These factors reduce encapsulation and particle surface roughness, avoiding moisture absorption [24]. The association of carbohydrates with proteins as wall materials corroborates the reduction in Tg temperature due to the formation of a protein film with a high glass transition temperature on the surface of the particles to be atomized. In this process, there is a decrease in particle interactions with the dryer chamber wall, with a consequent increase in product yield [26,27]. Furthermore, the protein film increases the emulsification capacity of the resulting powder in addition to promoting greater resistance to oxidation, contributing to the extension of the shelf life of the final product [28,29,30]. Three formulations containing WPI were prepared: Ma29W1 (maltodextrin 29% and WPI 1%), Ma20W10 (maltodextrin 20% and WPI 10%), and Ma15Me15W5 (maltodextrin 15%, methocel 10% and WPI 5%). The formulation M15Me15W5 (30.08%) had a yield comparable to the formulation Ma15Me15 (30.25%), while the formulations Ma29W1 and Ma20W10 presented better powder yields (43.33 and 46.51%, respectively). This result is of great interest, since WPI is a by-product of the cheese industry and its return to the production chain is also of great interest for increasing sustainability in the food industry. Furthermore, WPI is a protein-based carrier and increases the nutritional value of cocoa honey powder.

Atomization yields were below 50%, but the increase in the yield at this stage can be achieved with the application of dehumidified air, adjustments in formulations, and with use of custom-made industrial atomizers, which brings good prospects for the production of dehydrated cocoa honey on an industrial scale [31,32,33].

### 3.3. Characterization of Ingredients and Formulations

#### 3.3.1. Moisture Content, Total Solids, and Hygroscopicity

Some important parameters for the industrial development of new powdered foods regarding storage and use are low hygroscopicity, low moisture content, low degree of agglomeration, and high solubility in water [26]. In this context, several parameters of raw materials and products were evaluated. The moisture and solids content of cocoa honey and maltodextrin, methocel, and whey protein isolate used in the formulations were determined, and are presented in Table 2.

The moisture of the carriers is practically negligible in relation to that of cocoa honey. Total solids values were used to determine the yields of the powder formulations. The latter were characterized in terms of moisture content, water activity, and hygroscopicity, and the results of the physical analyses are presented in Table 3.

The physicochemical parameters determined for the cocoa honey powders did not show significant differences among the formulations (*p* < 0.05). Characteristics such as the presence of sugars, particles with larger sizes, and the porosity of the powder produced are factors that hinder the evaporation of water during the drying process [34]. The moisture content was between 11.24 and 11.72%, higher than the values reported for bee honey powder with the addition of rice vegetable protein (3.36 ± 0.20), pea vegetable protein (7.92 ± 0.04), and with both rice proteins and pea (6.82 ± 0.01) [35].

This parameter represents the composition of the water in a food system, but it is not sufficient to quantify the amount of water capable of favoring microbial development and influencing the quality of the product. Therefore, it is also desirable to determine the water activity (aW), to infer the quality, shelf life and food safety of the food [27,36]. The water activity (aW) determined for cocoa honey powders produced in this work ranged from 0.1464 to 0.1562. To prevent the growth of pathogenic microorganisms and improve stability, food products must have a low moisture content, and the dehydration process must be able to guarantee a water activity of less than 0.6 [37,38]. The aW values of the powders were below 0.1562, indicating that the cocoa honey powder formulations can be considered stable, since the biochemical reaction rates would be extremely slow due to the low aW found in the current work.

**Figure 2:**
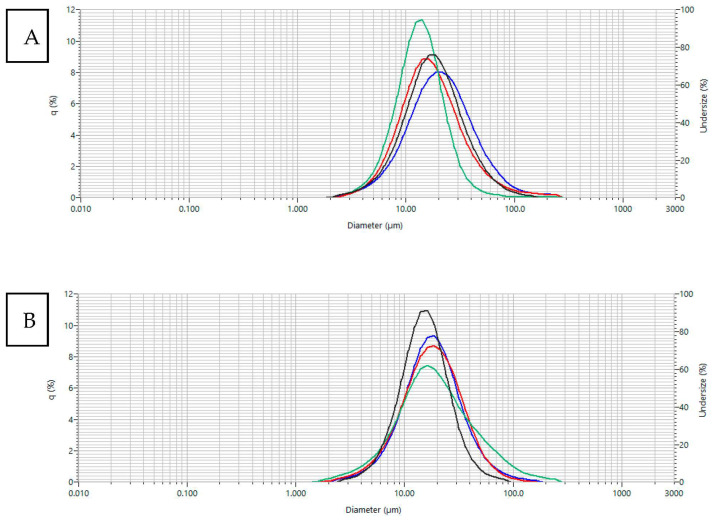
Particle size distribution for cocoa honey samples containing different excipients subjected to spray drying process. ■ Maltodextrin 15% + Methocel 15% (Ma15Me15); ■ Maltodextrin 29% + WPI 1% (Ma29W1); ■ Maltodextrin 20% + WPI 10% (Ma20W10); ■ Maltodextrin 15% + Methocel 10% + WPI 5% (Ma15Me10W5). (**A**) Before storage; (**B**) after 6 months of storage.

Hygroscopicity is also an important parameter related to the shelf life of new powdered foods, as it indicates the tendency of moisture absorption when the product is subjected to high relative humidity [39,40]. The hygroscopicity of cocoa honey powders ranged from 29.29 to 29.87 g of water/100 g of cocoa honey (Table 3), and the appearance of the formulations after the analyses is shown in Figure 2. These values are close to those reported for several dried sugary fruit-based products, such as blackberry powder (18.77–27.33%) [41] and rapeseed honey (22.0–27.4%) [42], and are better than the hygroscopicity of tamarind juice powder (46.03–56.32%) [26]. Lower hygroscopicity values of spray-dried sugar-rich foods are related to high glass transition temperatures.

#### 3.3.2. Particle Size and Dispersion Index

The degree of particle dispersion was evaluated taking into account the particle size (Table 4) and the particle size distribution of the various formulations (Figure 2). Particle size is an important parameter in dry materials, since the formation of particles with greater sizes during drying lead to a product with lower density and a more porous structure of the powder particles [24]. The span index was determined, as it is an indication of the poly dispersion of the particle size of the system, being represented by the poly dispersion index (PDI). The lower the span value, the narrower the particle distribution. The results indicated that the particle diameter corresponding to 90% of the accumulated distribution did not exceed 57 μm. The parameters referring to the particle size analysis presented in Table 4 show that the greatest dispersion of particles occurs in the Ma20W10 formulation with a span of 2.72, inferring greater variation in size between small (7.01 ± 0.06 μm), medium (18.25 ± 0.37 μm), and large (56.65 ± 1.17 μm) particles.

In the production of dehydrated foods, it is important to avoid the agglomeration of particles during rehydration or subsequent processing to incorporate the powdered material into other foods; therefore, the particles should be able to disperse completely in an aqueous medium. The particle size distribution for cocoa honey samples containing different excipients remained stable after 6 months of storage after being subjected to the spray-drying process, as shown in Figure 2. The presence of a single peak for all formulations is characteristic of particles of uniform or very close sizes (monomodal distribution) where most particles belong to a single fraction, without significant variations in size between them [43]. If there were systems containing groups of particles with different sizes or with concentration of either small or large particles, more than one peak would appear in the frequency distribution curve for particle classification, as a characteristic of multimodal distribution. The relationship between size and uniformity influences the solubility. In the monomodal distribution, solubility increases with decreasing particle size due to changes in surface area [44]. Therefore, the monomodal distribution favors the spontaneous solubility of cocoa honey powder in water systems.

**Figure 3:**
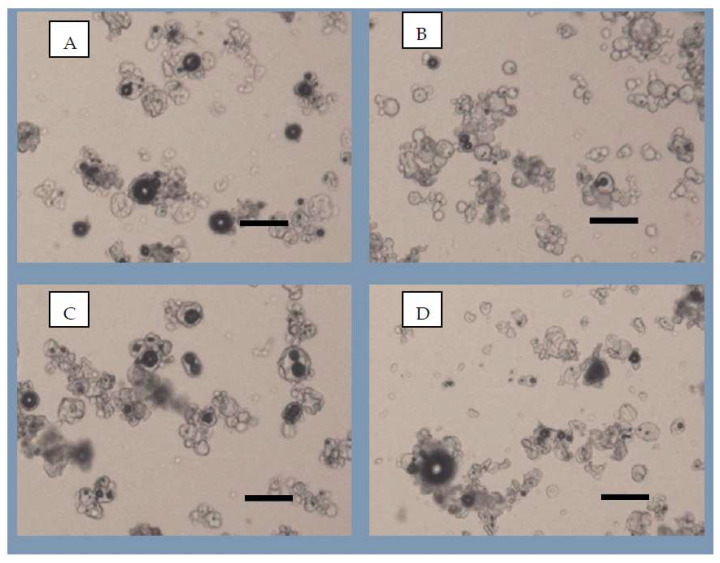
Images of the different formulations produced using optical microscopy after 6 months of storage (black line: 50 μm rule). (**A**) Maltodextrin 15%, methocel 15% (Ma15Me15); (**B**) maltodextrin 29%, WPI 1% (Ma29W1); (**C**) maltodextrin 20%, WPI 10% (Ma20W10); and (**D**) maltodextrin 15%, methocel 10%, WPI 5% (Ma15Me15W5).

Spray drying is advantageous because it generally produces homogeneous products from multicomponent solutions. Therefore, in addition to size, the surface morphology of microparticles is another important parameter related to powdered food ingredients. Figure 3 presents microscopic images after 6 months of storage, showing that all formulations contain spherical structures typical of materials subjected to atomization, which give them flow properties similar to fluids. This geometry makes post-drying operations such as filling, pressing, filtering and handling to incorporate the dried formulations in food processing easier and less expensive [45].

#### 3.3.3. Particle Morphology

The images show that the Ma15Me15, Ma29W1, and Ma15Me15W5 formulations presented amorphous particles far from each other, which reduces interparticle attraction and facilitates the dispersion of the powder in an aqueous medium (Figure 3A,B,D, respectively). When increasing the WPI concentration in the formulation, the particles tended to become larger and more grouped (Figure 3C), which corroborates the data presented in Table 4 for the Ma20W10 formulation, showing the formation of agglomerates. In this formulation, the powder was prepared with a higher percentage of the carrier WPI compared to the other formulations, implying that the increase in film efficiency is proportional to the increase in WPI concentration in the formulation [46]. It can also be observed that in formulations Ma15Me15, Ma20W10, and Ma15Me15W5 (Figure 3A,C,D, respectively), there is predominance of shapeless and heterogeneous particles. Rougher surfaces offer sites for microbial attachment and growth, which is undesirable due to the ease of contamination and consequent reduction in the product’s shelf life. In the Ma29W1 formulation (Figure 3B), the particles formed have smooth edges and a spherical shape, making this formulation more promising than the others.

#### 3.3.4. Interference of Atomization in Phenolic Constituents

Cocoa and its derivatives, such as chocolate, are known as important sources of food antioxidants due to their high content of polyphenol compounds [47]. The levels of polyphenols can be affected with processing, since high temperatures accelerate the degradation of antioxidants, which is undesirable because it would decrease the functionality of the final product. To evaluate this question, the total polyphenol contents for the powdered products obtained from the drying of cocoa honey were determined, and are presented in Table 5.

The polyphenol content of natural cocoa honey determined in this study was 47.9 ± 1.6 mg/100 g. The results (Table 5) show that, in the dry formulations, total polyphenol contents are significantly lower than the control cocoa honey. There is no significant difference in the phenolic contents for the formulations containing WPI (Ma29W1, Ma20W10, and Ma15Me15W5) (*p* < 0.05). As the contribution was not observed for the formulation containing only maltodextrin and methocel, the high polyphenol content in the formulations containing WPI is probably due to the antioxidant activity of this carrier [48]. This pattern is maintained after 6 months of storage. Thus, a new benefit related to the presence of WPI in formulations containing cocoa honey was evidenced.

It should be noted that the analysis of total polyphenols using the Folin–Ciocalteu spectrophotometric method is based on an oxidation–reduction reaction that is not exclusive to phenolic compounds. Thus, several compound samples such as ascorbic acid, reducing sugars, and tyrosine may interfere with the analysis, overestimating the polyphenols content present in a sample [49]. Although widely used for the prospecting the content of phenolic compounds in foods, this analysis presents an estimate of the total of polyphenols, and does not allow the perception of individual compounds.

The loss of phenolic components over time in cocoa powder honey formulations was evaluated (Table 5). It was found that the loss of polyphenols ranged from 6.04 to 10.67%, which can be considered low losses for a storage time of 6 months. A significant reduction (25 to 38%) in total phenolics was reported for cocoa pulp submitted to thermal processes at 85 °C for 60 s in relation to the pulp in natura, probably due to the adverse effects of heat, light, oxygen, and chemical reactions that take place during the storage. In addition, the degradation of the total phenolic compound content in pulps stored for eight weeks at temperatures of 4, 25, and 37 °C, was 14, 26, and 50% higher than non-treated pulp [6].

Several successful stabilized formulations have been reported with the use of WPI alone or in combination with other carriers with several matrixes, such as riboflavin, encapsulated in whey protein isolate [50], and black rice anthocyanins [51] with WPI and maltodextrin mixture.

## 4. Conclusions

The present work investigated the effect of maltodextrin, methocel, and whey protein isolate as carriers, in different proportions, on the spray drying processing of cocoa honey. The physicochemical, granulometric, and microscopic analyses adopted to characterize the resulting powders demonstrated that free-flowing powders were obtained successfully. The decrease in the water activity allows an increase in shelf-life, while the granulometry is in accordance with good powder dispersion capacity. Regarding carriers, WPI stood out for the production of cocoa honey powder, especially combined with maltodextrin. The increase in the proportion of WPI in the formulation improved production efficiency and reduced the hygroscopicity of the final product while enriching the powdered compound with proteins, therefore increasing its nutritional value. The formulation containing 29% maltodextrin and 1% WPI showed a greater retention of phenolic compounds naturally present in fresh cocoa honey, as well as predominance of cylindrical shaped particles compared to the other formulations. WPI has been shown to be a viable alternative for avoiding challenges often faced in the spray drying process, such as the formation of lumps on the drying chamber wall and the crystallization of low-temperature glass transition sugars, such as fructose. The quality of dry cocoa honey formulations using the atomization technique was also demonstrated by the maintenance of significant amounts of phenolic compounds. This product could be widely used in the food industry, as component of dry mixtures for various applications (cake preparations, powdered juices, cereal bars, jellies, drinks, confectionery, and desserts), as well as in nutraceutical products, opening up new commercial opportunities for producers and industries. However, more in-depth studies are necessary for the development of a product that can be effectively incorporated into the circular economy of cocoa production.

## Figures and Tables

**Table 1:** Composition of formulations containing cocoa honey and excipients.

Formulations	Description	Maltodextrin (g, dm)	Methocel (g, dm)	WPI (g, dm)
Ma30	Maltodextrin 30%	6.3	0	0
Ma15Me15	Maltodextrin 15% + Methocel 15%	3.15	3.15	0
Ma29W1	Maltodextrin 29% + WPI 1%	6.09	0	0.21
Ma20W10	Maltodextrin 20% + WPI 10%	4.2	0	2.1
Ma15Me10W5	Maltodextrin 15% + Methocel 10% + WPI 5%	3.15	2.1	1.05

WPI: whey protein isolate; dm: dry matter.

**Table 2:** Moisture content and total solids of the ingredients of the formulations.

	Cocoa Honey	Maltodextrin	Methocel	WPI
Moisture (g/100 g)	85.3 ± 0.20	2.9 ± 0.08	3.4 ± 0.01	1.8 ± 0.05
Total Solids (g/100 g)	14.7 ± 0.01	97.1 ± 0.10	96.6 ± 0.10	98.2 ± 0.11

Results expressed as mean of triplicate ± standard deviation.

**Table 3:** Results of physical analysis of cocoa honey samples dehydrated via spray drying using different carriers.

Formulations	Moisture (g/100 g)	Water Activity (aW)	Hygroscopicity
Ma15Me15	11.68 ± 0.29 ^a^	0.1562 ± 0.029 ^a^	29.67 ± 0.48 ^a^
Ma29W1	11.24 ± 0.23 ^a^	0.1489 ± 0.0031 ^a^	29.47 ± 1.84 ^a^
Ma20W10	11.72 ± 0.08 ^a^	0.1481 ± 0.0042 ^a^	29.29 ± 0.05 ^a^
Ma15Me15W5	11.61 ± 1.06 ^a^	0.1464 ± 0.0043 ^a^	29.87 ± 0.17 ^a^

Ma15Me15: maltodextrin 15%, methocel 15%; Ma29W1: maltodextrin 29%, WPI 1%; Ma20W10: maltodextrin 20%, WPI 10%; and Ma15Me15W5: maltodextrin 15%, methocel 10%, WPI 5%. aW: water absorbed/100 g sample. Results expressed as mean of triplicate ± standard deviation. Values followed by different lowercase letters are significantly different according to Tukey’s *t*-test (*p* < 0.05).

**Table 4:** Particle size distribution of cocoa honey formulations dried via spray drying containing different excipients.

	D10	D50	D90	PDI
	Average Diameter (µm)			
Ma15Me15	8.13 ± 0.06	17.88 ± 0.05	39.12 ± 0.29	1.73 ± 0.02
Ma29W1	7.72 ± 0.15	17.92 ± 0.26	38.88 ± 0.44	1.74 ± 0.04
Ma20W10	7.01 ± 0.06	18.25 ± 0.37	56.65 ± 1.17	2.72 ± 0.06
Ma15Me15W5	7.80 ± 0.02	15.22 ± 0.03	29.09 ± 0.09	1.40 ± 0.00

PDI: poly dispersion index; D10: particle diameter corresponding to 10% of the accumulated distribution; D50: particle diameter corresponding to 50% of the accumulated distribution; D90: particle diameter corresponding to 90% of the accumulated distribution span; calculated polydispersity index: (D90 − D10/D50). Results expressed as mean of triplicate ± standard deviation.

**Table 5:** Content of phenolic components over time in cocoa powder honey formulations.

Formulation	Polyphenol Contents (mg/100 g dm)		
	T0	T6	Loss (%)
Ma15Me15	329.71 ± 1.17 ^a^	294.53 ± 4.69 ^a^	10.67
Ma29W1	347.26 ± 1.30 ^b^	326.28 ± 4.22 ^b^	6.04
Ma20W10	423.75 ± 13.40 ^b^	383.73 ± 8.49 ^b^	9.44
Ma15Me15W5	434.07 ± 8.41 ^b^	390.88 ± 5.91 ^b^	9.95
Cocoa honey	424.3 ± 2.1 ^b^	389.0 ± 3.0 ^b^	8.32

dm: dry matter; T0: freshly prepared formulation; T6: formulation after 6 months of storage at room temperature (25 ± 3 °C). Results expressed as mean of triplicate ± standard deviation. Values followed by different lowercase letters are significantly different, according to Tukey’s *t*-test (*p* < 0.05).

## Data Availability

Data is contained in the article.
